# Bacteremic cholangitis due to *Raoultella planticola* complicating intrahepatic bile duct stricture 5 years post-laparoscopic cholecystectomy: a case report

**DOI:** 10.1186/s13256-021-02762-0

**Published:** 2021-04-07

**Authors:** David Blihar, Phenyo Phuu, Svetlana Kotelnikova, Edward Johnson

**Affiliations:** School of Medicine, St George University, True Blue, St. George, Grenada

**Keywords:** *Raoultella planticola*, Bacteremia, Cholangitis, Intrahepatic bile ducts, Gall bladder resection

## Abstract

**Background:**

*Raoultella Planticola* is a facultative anaerobic, gram-negative, water- and soil-dwelling rod bacterium rarely reported as a cause of human disease. However, the number of reported *R. planticola* infections is growing, without a concomitant increase in research on the microbe or its pathogenesis. Previous genomic studies demonstrating genetic similarities between *R. planticola* and *Klebsiella pneumoniae* suggest that capsule biosynthesis, mucoid phenotype, biofilm production, and lipopolysaccharide (endotoxin) synthesis may all be potential virulence factors of *R. planticola*. We present a unique case of *R. planticola* infection of the biliary tract 5 years after biliary surgery in a patient with no previously documented risk factors. We also use *in silico* techniques to predict virulence factors of *R. planticola*.

**Case presentation:**

This case report is the first to discuss a *R. planticola* infection in the biliary tract of late onset post-surgery (5 years) in a Caucasian patient with no previously documented risk factors.

**Conclusions:**

An in-depth search of the current literature did not yield other similar cases of *R. planticola* infections. Moreover, to the best of our knowledge, our case is the first case of *R. planticola* isolated from post-endoscopic retrograde cholangiopancreatography (ERCP) as part of biliary sepsis not associated with gastroenteritis. The late onset of the infection in our patient and the results of the *in silico* analysis suggest that *R. planticola* may have survived exposure to the host immune system through the creation of an intracellular biofilm or in a non-culturable but viable state (NCBV) for the 5-year period. The *in silico* analysis also suggests that biofilms, enterobactin, and mucoid phenotype may play a role in the pathogenesis of *R. planticola*. However, further research is needed to illuminate the significance of pili, capsule biosynthesis, and lipopolysaccharide (LPS) in the virulence of *R. planticola*. Lastly, as our patient did not have any risk factors previously associated with *R.* planticola, we suggest that biliary tract stricture, cholecystitis, and prior surgery may be possible novel risk factors.

## Background

The *Raoultella* genus comprises gram-negative, oxidase-negative, facultative anaerobic bacteria within the *Enterobacteriaceae* family. *R. planticola,* earlier known as *Klebsiella planticola* and *Klebsiella trevisanii*, is a gram-negative, rod-like bacterium first described by Ferragut [1] from aquatic and soil isolates and later differentiated from *Klebsiella* after phylogenetic analysis by Drancourt and associates [2]. Matrix-assisted laser desorption/ionization–time of flight (MALDI-TOF) mass spectrometry is the current method used to identify and differentiate *R. planticola* [3].

*R. planticola* is an emerging pathogen which has been linked to fatal infections. Only 33 cases of *R. planticola* were reported prior to the middle of 2015 [4]. There have been 19 novel cases reported since that time. Pediatric cases, although extremely rare, have also been reported [5]. Further, *R. planticola* may cause bacteremia, pneumonia, intra-abdominal infections, urinary tract infections, soft tissue infections, and conjunctivitis [6–8]. To our knowledge, there have been no reports of *R. planticola* biliary tract infections or a thorough investigation of microbial virulence or immune escape. Herein, we report a case of *R. planticola* biliary tract infection as a long-term postsurgical complication in a 31-year-old woman who initially presented with acute cholecystitis. We also discuss the results of a genome-based comparison between *R. planticola* and *K. pneumoniae* in order to examine possible virulence factors of *R. planticola*.

## Case presentation

A 31-year-old Caucasian woman presented to the emergency department with sudden onset of abdominal pain, fever, chills, and malaise. She had a history of laparoscopic cholecystectomy in 2008 complicated by bile leak requiring biliary stents. In 2011, she developed hepatic cysts, which were surgically extirpated in 2011 and 2012. She remained afebrile and mostly asymptomatic, with only occasional mild right upper quadrant pain until the current presentation which caused her to seek medical attention at the emergency department. Importantly, she denied any history of solid organ transplants, hematologic malignancy, chemotherapy, transplantation neutropenia, cirrhosis, seafood ingestion, or proton pump inhibitor (PPI) use.

**Fig. 1:**
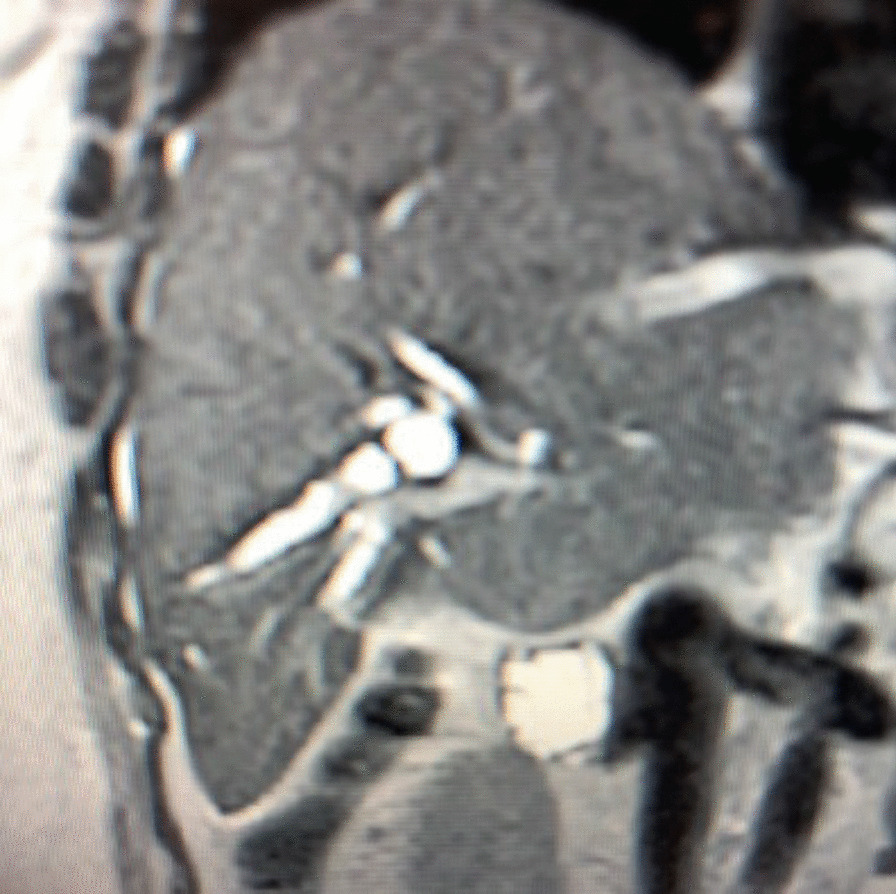
Magnetic resonance cholangiopancreatography dextral sagittal view of the abdomen

**Fig. 2:**
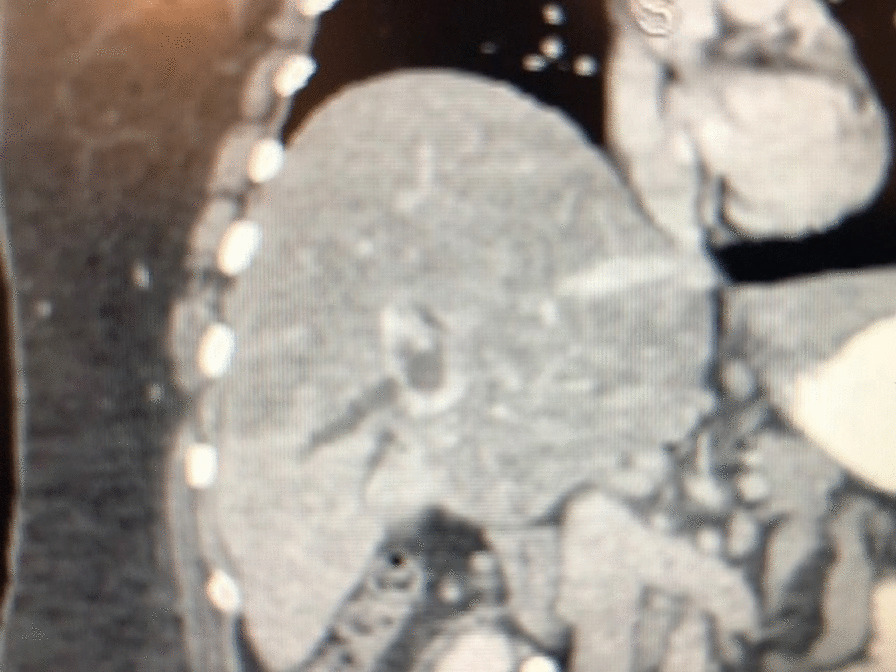
Magnetic resonance cholangiopancreatography posteroanterior view of the abdomen

**Fig. 3:**
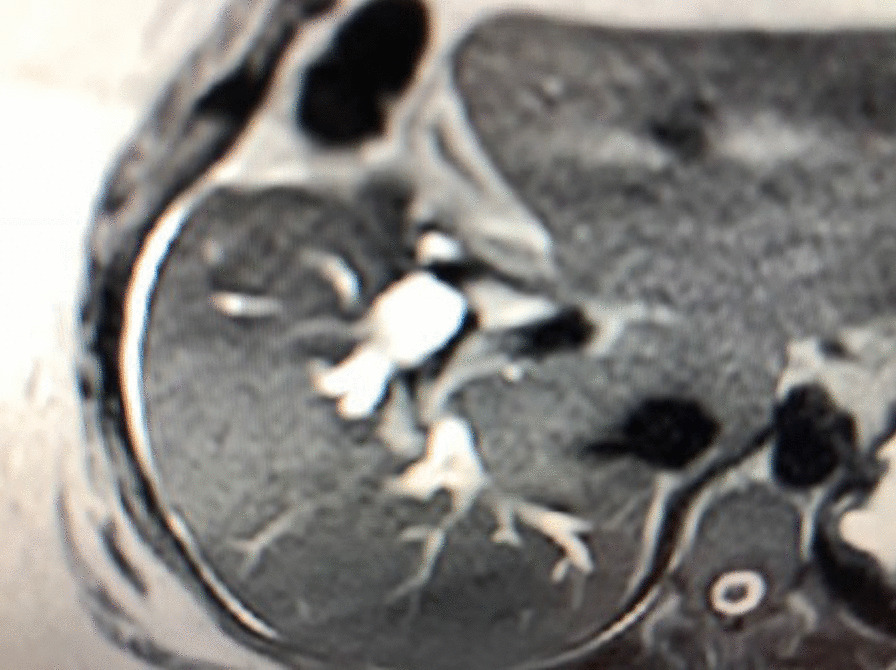
Magnetic resonance cholangiopancreatography sinistral sagittal view of the abdomen

Vital signs on presentation were temperature of 98.1 °F, heart rate of 85 beats per minute, respiration of 18 breaths per minute, and blood pressure of 126/83 mmHg. Physical examination revealed an afebrile, anicteric female in moderate, painful distress with slight, diffuse abdominal tenderness on palpation. Laboratory/radiographic tests revealed a white blood cell count of 41.1 cells/μL; elevated liver enzymes (alanine aminotransferase 102 U/L and aspartate aminotransferase 74 U/L); alkaline phosphatase of 318 U/L; and total bilirubin of 2.4 mg/dl. Computed tomography and magnetic resonance cholangiopancreatography (MRCP) were significant for dilated right intrahepatic bile duct with evidence of a surgically absent gall bladder (Figs. 1, 2, 3). During admission in the emergency room, the patient became febrile, and blood cultures (BC) were drawn. In light of the clinical picture and imaging studies, biliary sepsis and bacteremia due to intrahepatic duct stricture were suspected. The patient was admitted to the hospital and empirically started on piperacillin/tazobactam. The BC was positive for gram-negative rods in two of two peripheral BC after 24 hours. *R. planticola* was reported as the isolate on the third hospital day and was resistant to ampicillin and piperacillin but susceptible to ceftriaxone (microbial resistance and susceptibilities were completed by Quest Diagnostics). Therapy was changed to ceftriaxone 2 g parenterally every 24 hours, and the patient quickly improved clinically, with normalization of liver function within 3 days (hospital day 6). She was discharged on home therapy with referral for subsequent evaluation and treatment of her intrahepatic duct strictures.

## Case discussion and methods

### Previously reported *R. planticola* cases

**Fig. 4:**
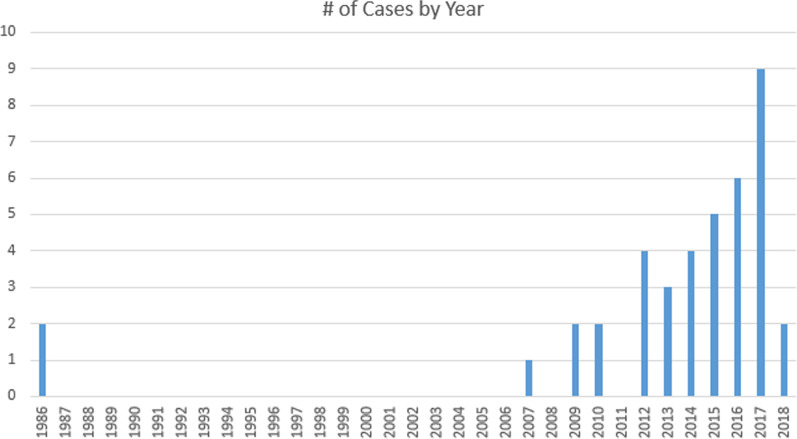
Timeline of reported cases associated with isolation of *R. planticola*

*Raoultella planticola* is an emerging bacterial pathogen (see Fig. 4) that has previously been associated with nonclinical environments such as aquatic habitats and therefore has been linked to consumption of seafood [9]. However, case studies since 1985 have indicated an increased number of clinical cases [10] and multiple organ infections [11]. Previously, *R. planticola* was isolated from patients with comorbid leptospirosis [10] and found to be a cause of pneumonia [12]. *R. planticola* was also observed to occur in hematological malignancy when the organism was isolated from the oral ulcers of a patient with chemotherapy-induced oral mucositis [13]. Similarly, other cases have been documented that suggest an increased susceptibility to infection in immunocompromised states [13]. Table 1 summarizes all reported *R. planticola* infections prior to 2018 found during an in-depth literature search.

**Table 1:** A summary of the epidemiology of *R. planticola* cases

Reported case	Clinical manifestation	Culture site	Age	Sex (M/F)	Region	Outcome
[35]	Bacteremia	Blood	69	Unknown	France	Recovered
[35]	Pneumonia	Blood, sputum	57	Unknown	France	Recovered
[36]	Pancreatitis	Peritoneal fluid	45	M	Brazil	Recovered
[37]	Pneumonia	Blood	83	F	Ohio, USA	Died
[37]	Soft tissue	Blood	64	M	New Jersey, USA	Died
[38]	Cellulitis	Wound	30	M	Ireland	Recovered
[39]	Soft tissue	Unknown	66	M	Texas, USA	Unknown
[40]	Cholangitis	Blood	65	M	Japan	Improved, transferred
[41]	Necrotizing fasciitis	Abdominal fluid	66	M	South Korea	Recovered
[42]	Cholecystitis	Gallbladder Fluid	62	F	UK	Recovered
[43]	Cholangitis	Blood	59	M	Ontario, Canada	Recovered
[44]	UTI	Urine	89	M	New Mexico, USA	Recovered
[45]	Bacteremia	Blood	63	M	Spain	Recovered
[46]	Prostatitis	Urine	67	M	Greece	Recovered
[47]	Bacteremia from seafood	Blood	56	F	Ontario, Canada	Recovered
[7]	Conjunctivitis	Conjunctival swab	58	F	UK	Recovered
[48]	Cholangitis	Blood	70	M	Italy	Recovered
[49]	Cholecystitis	Biliary fluid	49	M	Connecticut, USA	Recovered
[50]	Cholangitis	Unknown	Unknown	Unknown	Unknown	Unknown
[6]	Pneumonia	Sputum	60	M	China	Died
[51]	UTI	Urine	92	F	Connecticut, USA	Recovered
[52]	Cystitis	Urine	1	M	South Korea	Recovered
[53]	Peritonitis	Peritoneal fluid	65	M	South Korea	Recovered
[12]	Pneumonia	Sputum	58	M	South Korea	Recovered
[8]	Conjunctivitis	Conjunctival swab	88	F	Malta	Recovered
[8]	Conjunctivitis	Conjunctival swab	71	M	Malta	Unknown
[8]	Conjunctivitis	Conjunctival swab	15	F	Malta	Unknown
[8]	Conjunctivitis	Conjunctival swab	69	F	Malta	Recovered
[54]	Prostatitis	Prostatic fluid	53	M	New York, USA	Recovered
[55]	UTI	Urine	57	M	Unknown	Recovered
[56]	Implantation site infection	Pus from site	79	M	Unknown	Recovered
[57]	UTI	Urine	73	M	Florida	Recovered
[58]	UTI	Urine	2 months	F	Unknown	Recovered
[59]	UTI	Urine	57	M	Unknown	Recovered
[60]	Cirrhosis	Blood	66	M	Unknown	Recovered
[13]	Oral mucositis	Oral ulcers	16	M	Unknown	Recovered
[61]	Spinal epidural abscess	Unknown	Unknown	Unknown	Unknown album	Recovered
[62]	Wound infection	Unknown	73	F	Unknown	Recovered
[5]	Conjunctivitis	Conjunctival swab	28 weeks	F	Unknown	Recovered

*UTI* urinary tract infection, *M* male, *F* female

Analysis of all the documented patients showed that *R. planticola* caused bacteremia in 22% of cases, soft tissue infections in 17%, urinary tract infections in 15%, lower respiratory tract infections in 10%, and eye infections in 10%. Sources of isolation correlated with the infected organ system (*r*^2^ = 0.72). The annual timeline frequency of documented infections caused by this pathogen potentially indicate a biannual prevalence (see Fig. 4).

### Pathogenesis and virulence factors in *R. planticola* genome

The pathogenesis of *R. planticola* has not yet been established; however, fimbria, biofilm production, encapsulation, lipopolysaccharide (LPS), and siderophores have been observed to be important virulence factors in the closely related species *Klebsiella pneumoniae* species [3, 14].

In order to investigate possible virulence factors for *R. planticola*, *in silico* analysis was conducted using the National Center for Biotechnology Information (NCBI) Basic Local Alignment Search Tool (BLAST) (NCBI.gov) [15], Universal Protein Resource (UniProt; uniprot.com) [16], and Integrated Microbial Genomes (IMG; img.jgi.gov) system [17]. In the analysis, previously documented virulence factor gene sequences, including known *K. pneumoniae* virulence factor gene sequences, were used to generate queries to blast against the *R. planticola* genome. Using these databases, gene sequences and accession numbers for virulence factors in *K. pneumoniae* were found for over 40 genes pertaining to pili components, pili chaperone proteins, biofilm synthesis and initiation, capsule assembly, capsule biosynthesis, capsule initiation, LPS synthesis, outer membrane surface protein chaperones, and expression of mucoid phenotype (genes present, see Tables 2, 3). Using the gene function on IMG [17], permanent drafts of *K. pneumoniae* pKP469IL Plasmid (B) [P], *K. pneumoniae* pKP531IL (B) [P], *K. pneumoniae* 1162281 (B) [P], and *K. pneumoniae* 1191100241 (B) [P] were searched for gene sequences and accession numbers for known virulence factors [18]. The genome for *R. planticola* strain GODA was selected as a target for querying the virulence factors relevant for endotoxins, capsules, fimbria, pili production, and biofilm production using the BLASTn and BLASTp tools available from NCBI.gov [15]. In order to investigate the clinical relevance, the genome of ATCC 33531 [19] was compared against GODA and found to be 99% identical (see Table 4).

**Table 2:** Genomic identification of virulence factors in the genome of *R. planticola* ATCC 33531

Genes present in *Raoultella Planticola* (Accession CP 019899.1)		Blast results against *R. Planticola* genome				
Gene name	Gene ID	Max score	Total score	Query cover (%)	E-value	Identity (%)
LPS biosynthesis protein WzzE	NC_016845.1	28696	4.24E+06	71	0.0	93
O-acetyltransferase	NC_016845.1	2143	2143	67	0.0	82
Major type 1 subunit fimbria	2546382621		No significant similarity found			
Pilin (type 1 fimbria component protein)	2546385281		No significant similarity found			
KpsS (capsule synthesis)	NC_025184.1	2146	2.01E+04	5	0.0	99
Uncharacterized protein related to capsule biosynthesis enzymes	NC_010870.1	2100	10206	1	0.0	99
Capsule assembly protein Wzi	2549022389	950	950	94	0.0	79
Periplasmic chaperone for outer membrane proteins Skp	2546385958	693	693	100	0.0	92
Periplasmic chaperone for outer membrane proteins SurfA	2546385592	1474	1474	100	0.0	87
Regulator of mucoid phenotype rmpA	2657583		No significant similarity found			
Regulator of mucoid phenotype rmpA2	2657677		No significant similarity found			
Putative negative regulator of RcsB-dependent stress response	2549023015	538	538	100	3.00E-153	82
Regulator of capsule synthesis rcsA	CIG23_03380	*299*	299	100	1.00E-93	69

*A* adenine,* T* thymine,* C* cytosine,* G* guanine

**Table 3:** The results of queries previously functionally annotated in *R. planticola*

Functionally annotated genes in *R. planticola* (Accession CP 019899.1)	
Gene name	Gene ID
Biofilm protein TabA	EG12530 tabA
Biofilm regulator BssS	UA70_04275
Biofilm formation protein BSSR	UA70_18665
Type 1 fimbriae regulatory protein FimB	2588758217
Type 1 fimbriae regulatory protein FimE	2588758216
Fimbrial, FimD or usher-like	2588757070
Surface assembly of capsule Wzi	IPR026950
ybdA enterobactin exporter	2588761180
Enterobactin synthetase component D	2588761188
Enterobactin synthetase component F	2588761184

TabA is a gene responsible for initiation of biofilm synthesis [63]. BssS and BssR are genes associated with biofilm stress response induction and upregulation of motility transcription [64]. FimB, FimD, and FimE are genes encoding chaperone proteins for pili components [65]. Wzi is a gene associated with the synthesis of the capsule [24]. Enterobactin synthetase components D and F and ybdA enterobactin exporter are genes involved in the production of the siderophores enterobactin which allow the pathogen to outcompete the host iron-acquisition system [14]

**Table 4:** Information for the host-associated isolated ATCC 33531 strain and the environment-isolated GODA strain

Species	Strain	Accession no.	Ecosystem	Chromosomal cassette gene %		*In silico* genome hybridization			
*R. planticola*	GODA	CP019899.1	Soil and ground water	Not previously documented	Max score	Total score	Query cover %	E value	Identity %
*R. planticola*	ATCC 33531	CP023874.1	Host-associated	98.23	5.12E+05	1.03E+07	93	0	99

For functional gene annotation, greater than 60% query coverage, 70% nucleotide identity, and Expect (E)-value below 0.001 were used as the minimum similarity criteria between functionally documented genes of *K. pneumoniae* and unknown *R. planticola* genes (see Tables 2, 3). Novel queries identified by BLASTn analysis of *R. planticola* GODA against *K. pneumoniae* are shown in Table 2, and previously functionally annotated queries are shown in Table 3.

## Results and discussion

The results of blasting for capsule and mucoid production genes are shown in Tables 2 and 3.

### Endotoxin production

*Wzze* [20] and O-acetyltransferase [21], genes known to be involved in the synthesis of LPS, showed 93% and 82% identity in 67% and 71% of the gene fragments, respectively (see Table 2). This indicates that those mechanisms might be shared between *R. planticola* and *K. pneumoniae* and that *R. planticola* may produce LPS endotoxin, which could explain how it causes a bacteremia, avoids immune response, or causes sepsis.

### Capsule and mucoid production

We observed the following virulence factor relationships between the target genomes: Wzi (99% identity in 94% of the gene fragments); rcsA (69% identity in 100% of the gene fragments); rcsB (82% identity of 100% of the gene fragments); KpsS (99% identity in 5% of the gene fragments) suggesting a shared prosthetic group; uncharacterized protein (99% identity in 1% of the gene fragments) suggesting a shared prosthetic group. The presence of such genetic similarities indicates that *R. planticola* has the potential to synthesize, regulate, and assemble a capsule and express a mucoid phenotype that could help it escape the host immune system [22–26]. Mucoid phenotype regulators rmpA and rmpA2 did not show significant matching, indicating that capsular mucoid composition in *R. planticola* may be expressed through a different mechanism from that in *K. pneumoniae*. The results of blasting for capsule and mucoid production genes are shown in Tables 2 and 3.

### Adhesins, pili, and fimbria

*Skp* and *SurfA* queries were found to be 92% and 87% identical, respectively, between *R. planticola* and *K. pneumoniae*, suggesting that *R. planticola* codes for periplasmic chaperoning of outer membrane protein assembly [27, 28]. Type 1 fimbria regulatory proteins *FimB* and *FimE* and fimbria *FimD* queries have previously been documented in *R. planticola* (see Table 3). No other similarities for different fimbrial components were found. This variability that *R. planticola* possesses in its fimbria may contribute to its host cell attachment and opsonization prevention in ways that differ from those mechanisms in *K. pneumoniae*.

### Biofilm production

*Raoultella planticola* was found to possess genes similar to *K. pneumoniae* that have previously been shown to cause infection via biofilm production, upregulation of motility factors, outcompeting host cells for iron, and mucous production (see Table 3).

### Gene cassettes

Whole genome blasting analysis between host-associated ATCC 33531 and GODA was conducted to identity chromosomal cassettes. We observed identity of 99% in 93% of gene fragments, suggesting high gene density that could allow efficient regulation of gene expression in mechanisms of antibiotic resistance, host immune system evasion, host cell attachment and invasion, and intracellular survivability (see Table 4) [29]. *R. planticola* may contain pathogenicity islands, a potential result of transduction that would also be involved in bacterial adaption, but further research is needed to confirm this.

### Organ systems affected, virulence factors, and potential latent infection in our case

An in-depth search of the current literature did not yield other case studies with a similar isolation of *R. planticola*. We were also unable to identify another case of *R. planticola* isolated after endoscopic retrograde cholangiopancreatography (ERCP) as part of biliary sepsis not associated with gastroenteritis. Further, a detailed history and chart review of our patient did not show any of the previously reported risk factors associated with *R. planticola* including bacteremia/sepsis of the gastrointestinal tract (GI), biliary malignancy, chemotherapy, transplantation, neutropenia, cirrhosis, seafood ingestion, or PPI usage. It is possible that our patient had recently become infected with *R. planticola* rather than during the time of her laparoscopic procedure; however, as discussed above, an extensive attempt to document any previously associated risk factors failed to illuminate any. It has been reported that patients with chronic biliary strictures are at increased risk of cholangitis, possibly due to static biliary fluid in the stenotic biliary system or because of abnormal anatomic morphology that facilitates bacterial adhesion and colonization [30–32].

Therefore, we speculate that our patient’s bacteremia, which developed 5 years postoperatively, may be due to possible latency of the pathogen. The *in silico* results might also indicate that this organism survived exposure to the host immune system through the employment of an intracellular biofilm or in a non-culturable but viable (NCBV) state for the 5-year period [33, 34]. Our results also suggest that biofilms, enterobactin, and mucoid phenotype are likely virulence factors in the pathogenesis of *R. planticola’s* ability to cause infection. Additionally, we identified a conservation of genes involved in pili synthesis regulation, fimbrial protein chaperoning, capsule biosynthesis, and endotoxin production; however, the genetic variation of genes coding for pili, fimbria, and capsule polysaccharide composition may indicate that these genes are subject to antigenic variation or reductive evolution in an attempt to avoid the host immune system. Multiple genomes of newly isolated clinical *P. planticola* should be sequenced in order to evaluate its level of evolutionary conservation of the extracellular and surface glycoproteins.

## Conclusions

This unique case adds to the literature on the GI affinity of *R. planticola* and, with the results of the *in silico* analysis, suggests that potential novel risk factors for infection may be biliary tract stricture, cholecystitis, and prior surgery.

## Data Availability

No data or samples from the patient will be made available due to patient privacy.

## Abbreviations

BC: Blood cultures
ERCP: Endoscopic retrograde cholangiopancreatography
GI: Gastrointestinal tract
LPS: Lipopolysaccharide
MALDI-TOF: Matrix-assisted laser desorption/ionization–time of flight
MRCP: Magnetic resonance cholangiopancreatography
NCBV: Non-culturable but viable state
PPI: Proton pump inhibitor
